# Identification, Characterization and Application of a G-Quadruplex Structured DNA Aptamer against Cancer Biomarker Protein Anterior Gradient Homolog 2

**DOI:** 10.1371/journal.pone.0046393

**Published:** 2012-09-28

**Authors:** Jie Wu, Chi Wang, Xilan Li, Yanling Song, Wei Wang, Cong Li, Jia Hu, Zhi Zhu, Jiuxing Li, Weiyun Zhang, Zhongxian Lu, Chaoyong James Yang

**Affiliations:** 1 State Key Laboratory of Physical Chemistry of Solid Surface, Key Laboratory for Chemical Biology of Fujian Province, Key Laboratory of Analytical Sciences, and Department of Chemical Biology, College of Chemistry and Chemical Engineering, Xiamen University, Xiamen, People's Republic of China; 2 Xiamen City Key Laboratory of Metabolism Disease, School of Pharmaceutical Sciences, Xiamen University, Xiamen, China; Institute of Cancerology Gustave Roussy, France

## Abstract

**Background:**

Anterior gradient homolog 2 (AGR2) is a functional protein with critical roles in a diverse range of biological systems, including vertebrate tissue development, inflammatory tissue injury responses, and cancer progression. Clinical studies have shown that the AGR2 protein is overexpressed in a wide range of human cancers, including carcinomas of the esophagus, pancreas, breast, prostate, and lung, making the protein as a potential cancer biomarker. However, the general biochemical functions of AGR2 in human cells remain undefined, and the signaling mechanisms that drive AGR2 to inhibit p53 are still not clearly illustrated. Therefore, it is of great interest to develop molecular probes specifically recognizing AGR2 for its detection and for the elucidation of AGR2-associated molecular mechanism.

**Methodology/Principal Findings:**

Through a bead-based and flow cytometry monitored SELEX technology, we have identified a group of DNA aptamers that can specifically bind to AGR2 with *K_d_* values in the nanomolar range after 14 rounds of selections. Aptamer C14B was chosen to further study, due to its high binding affinity and specificity. The optimized and shortened C14B1 has special G-rich characteristics, and the G-rich region of this binding motif was further characterized to reveal an intramolecular parallel G-quadruplex by CD spectroscopy and UV spectroscopy. Our experiments confirmed that the stability of the G-quadruplex structure was strongly dependent on the nature of the monovalent ions and the formation of G-quadruplex structure was also important for the binding capacity of C14B1 to the target. Furthermore, we have designed a kind of allosteric molecule beacon (aMB) probe for selective and sensitive detection of AGR2.

**Conclusion/Significance:**

In this work, we have developed new aptamer probes for specific recognition of the AGR2. Structural study have identified that the binding motif of aptamer is an intramolecular parallel G-quadruplex structure and its structure and binding affinity are strongly dependent on the nature of the monovalent ion. Furthermore, with our design of AGR2-aMB, AGR2 could be sensitively and selectively detected. This aptamer probe has great potential to serve as a useful tool for early diagnosis and prognosis of cancer and for fundamental research to elucidate the biochemical functions of AGR2.

## Introduction

Anterior gradient homolog 2 (AGR2) was identified initially as a secretory factor expressed in the anterior region of the dorsal ectoderm in Xenopuslaevis embryos, where it was postulated to mediate the specification of dorsoanterior ectodermal fate, particularly in the formation of the cement gland [1]. Clinical studies have further shown that the AGR2 protein is overexpressed in a wide range of human cancers, including carcinomas of the esophagus, pancreas, breast, prostate, and lung [2]–[6]. More biological studies in these cancer cell lines have indicated a significant role for AGR2 in tumor-associated pathways, including tumor growth, cellular transformation, cell migration, limb regeneration, and metastasis [5], [7]–[9]. However, the general biochemical functions of AGR2 in human cells remain undefined, and the signaling mechanisms that drive AGR2 to inhibit p53 are still not clearly illustrated [10]. Therefore, the development of molecular ligands specifically recognizing AGR2 is of great significance to early diagnosis and prognosis of cancer and to fundamental research for the elucidation of the biochemical functions of AGR2.

Various ligands have been developed for specific molecular recognition, such as small molecules, antibodies, and peptides [11]–[13]. More recently, another type of molecular ligand, named aptamer, has drawn significant attention. Aptamers, single-stranded modified or unmodified oligonucleotides (RNA or DNA), are generated through *in vitro* selection process or SELEX (Systematic Evolution of Ligands by EXponential enrichment) with high binding affinity and specificity towards defined targets [14], [15]. The selected aptamers can recognize a wide variety of targets, including small molecules, proteins, cells and tissues relying on their diverse tertiary structures. Compared to antibodies, aptamers have low molecular weight, fast tissue penetration rate, high stability and low immunogenesis [16]. They can be chemically synthesized with low cost and modified easily with various reporters [17]. Furthermore, they can be ligated and/or amplified by enzymes *in vitro* [18]. These advantages make aptamers promising ligands for medical and pharmaceutical research, such as drug development, disease diagnosis, and targeted therapy [19].

The possibilities provided by aptamers are enormous, and some aptamers have already shown many important applications in bioanalysisand biomedicine [20]–[23]. Particularly, several aptamers have been generated against cancer-related proteins, such as PDGF, VEGF, HER3, NFkB, tenascin-C, or PMSA [24]–[26]. Many aptameric sensors, probes and assays have been developed to allow sensitive and selective detection of these cancer biomarker proteins [27]. For instance, Yang *et al* has reported a light-switching excimer aptamer probes for sensitive quantitative detection of PDGF in cell media [28]. Kwon *et al* have developed a functionalized polypyrrole nanotube with aptamer to build a VEGF biosensor [29]. Aptamers have also been applied for molecular imaging to *in vivo* characterize the complex pathogenic activities that accompany tumor growth for disease early diagnosis and pathogenesis measurement [30]–[33]. Since the targets for aptamers could be intracellular, extracellular or cell-surface biomolecules, various therapeutic methods have been developed using the aptamers as targeting reagents [34]–[37], which greatly broaden the range of targeted therapy. In addition, some therapeutically useful aptamers have been found to inhibit protein–protein interactions, such as receptor–ligand interactions, and thereby function as antagonists [38].

In this study, using the bead-based and flow cytometry monitored SELEX technology, we aimed to obtain specific aptamers to AGR2 and study theirs structure and potential function. Beads-based SELEX allowed the use of simple, yet effective, flow cytometry analysis to monitor the progress of the selection, avoiding the tedious, time consuming and radioactive EMSA process [39]–[43]. After 14 rounds of selection, we have identified a group of DNA aptamers that specifically bound to AGR2 with high affinities. Structural studies on one of the aptamer sequences, C14B, revealed an intramolecular parallel G-quadruplex, and its structure and binding affinity to AGR2 depend on K^+^ ion intensively. Furthermore, we designed an allosteric molecule beacon AGR2-aMB based on the identified aptamer, which enables simple, sensitive and selective detection AGR2. The aptamer sequences and AGR2-aMB reported in this study are potentially useful tools for early diagnosis and prognosis of cancer and for fundamental research to elucidate the biochemical functions of AGR2.

## Results and Discussion

### Selection of DNA aptamers to recognize AGR2

**Figure 1:**
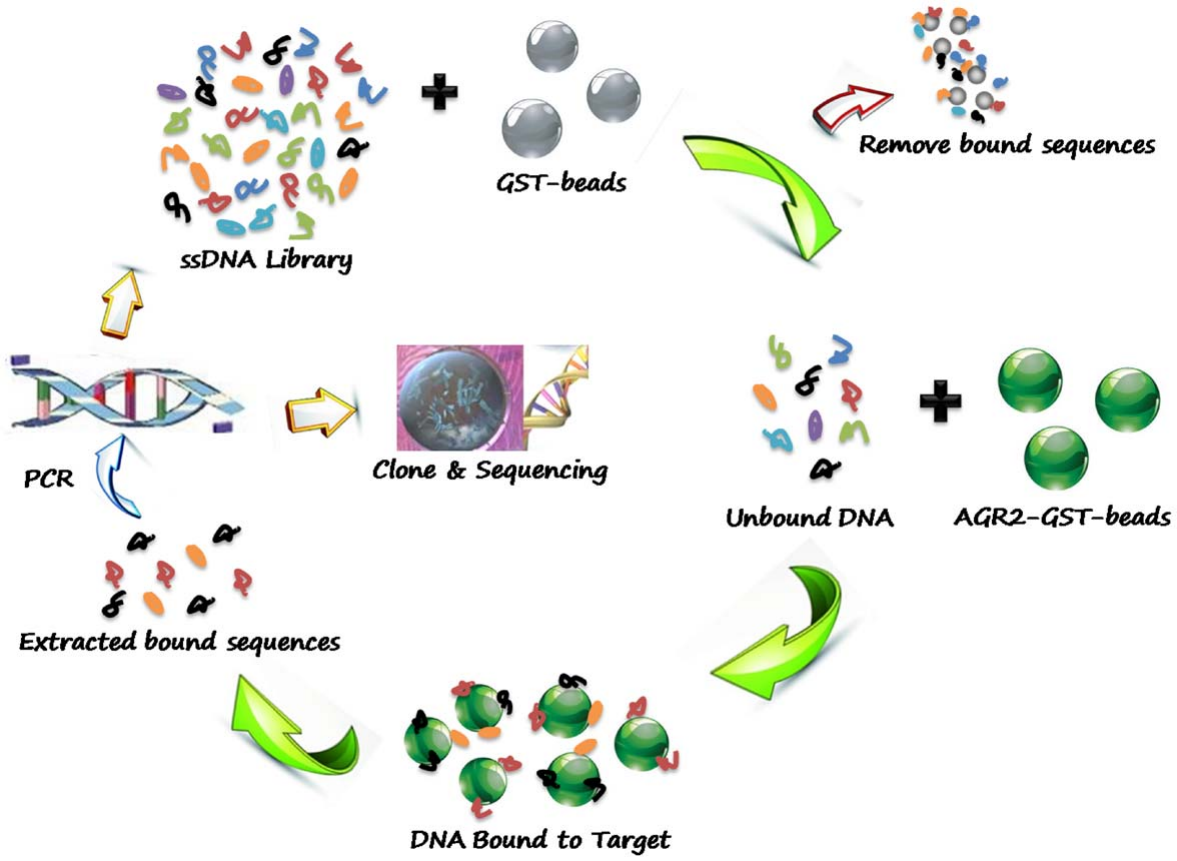
Schematics of systematic evolution of DNA aptamers against AGR2. In sepharose-beads-based SELEX process for protein AGR2, the GST-beads were incubated with the ssDNA library for counter-selection to remove nonspecific sequences. The unbound DNAs were then incubated with AGR2-GST-beads for target-selection. After harshly washing, the AGR2-specific DNA sequences were subsequently amplified by PCR for the next round of selection, or for cloning and sequencing to identify individual aptamers after flow cytometry analysis.

To identify aptamers against AGR2, recombinant AGR2 was fused with glutathione-S-transferase (GST) to facilitate the attachment of the protein to solid supports (Sepharose GSH-beads). The resulting AGR2-GST-beads were used as the positive target in SELEX while the GST-beads as negative control to remove non-specific surface binding sequences. The process of *in vitro* sepharose-bead-based SELEX is schematically illustrated in Figure 1.

**Figure 2:**
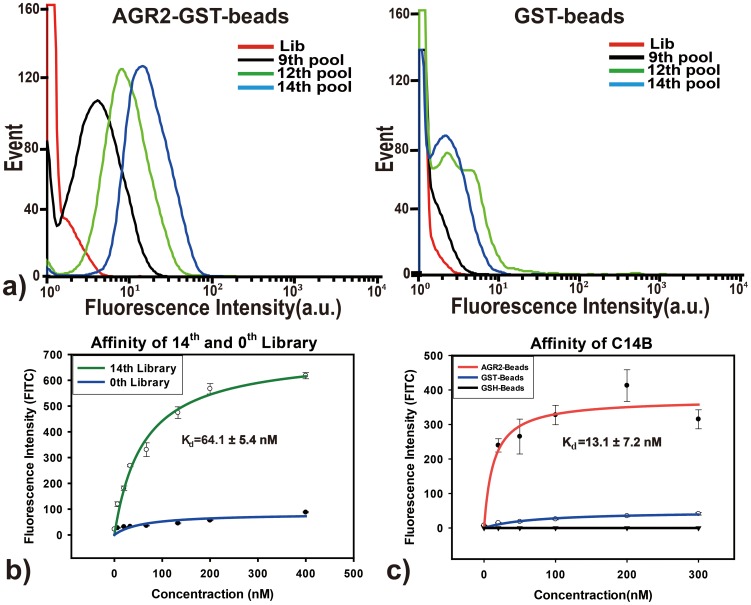
Enrichment of DNA sequences that bind to AGR2-GST-beads. a) Flow cytometry assay to monitor the binding of a selected pool with AGR2 (target protein) and GST (control protein). The red curve represents the background binding of unselected library. For the target protein AGR2, there was an increase in binding capacity of the pool as the selection progressed, while there was no observable change for the control protein GST. The final concentration of the selected pool in binding buffer was 100 nM. b) Determination of the dissociation constant of the enriched library and unselected library to AGR2. c) Fluorescence measurements to determine the dissociation constant of selected aptamer C14B. Two negative controls, GST-beads and GSH-beads, were carried out. The data were the average of triplicate experiment results.

An 87-nucleotide (87-nt) single-stranded DNA (ssDNA) library with 45 random bases flanked by two primer sequences (22-nt and 20-nt) was subjected to the SELEX procedure. The library was first allowed to interact with excess negative control beads, and only the DNA sequences that did not bound to the GST-beads were collected. The collected sequences were then incubated with AGR2-GST-beads. After rigorous washing, those sequences that either did not bind, or only weakly bound to the target were discarded. Only the sequences that bound strongly enough were retained on beads, and the bead-ssDNA complexes were collected and amplified by PCR for the next round of selection. After multiple rounds of selection, the subtraction process efficiently reduced the DNA sequences that bound to the GST beads, while those AGR2-specific aptamer candidates were gradually enriched. The progress of the selection process was monitored by flow cytometry. The stronger binding of DNA library to AGR2, the more FAM labeled sequences bound to the beads, thus the higher fluorescence intensity the beads would emit. With the increasing number of selection cycles, steady increases in fluorescence intensity on the target beads were observed (Figure 2a). The binding affinity of the enriched library after 14 rounds of selection was determined to be in the nanomolar range (K_d_ = 64.1±5.4 nM), while there was no observable binding of the library to control beads (Figure 2b). These results suggested that the DNA aptamers specifically recognizing AGR2 were enriched during the selection process.

### Characterization of selected aptamer sequences

After 14 rounds of selection, the enriched DNA pool was cloned and sequenced. The sequencing data for clones were analyzed by using sequencing analysis software Clustal W 6.0 [44]. The sequences were grouped based on the homology similarity of the DNA sequences from individual clone. Among the sequences from 62 clones, there were two subfamilies each containing multiple sequences with common features. One subfamily is guanosine-rich sequences (22 clones), and the other is thymine-rich sequences (40 clones) (Figure S1). Four sequences were chosen and synthesized for further characterization (Table S1): three sequences from G-rich subfamily, C14A (appeared 2 times), C14B (appeared 3 times) and C14C (appeared 5 times), and one sequence C14D (appeared 2 times) from T-rich subfamily. Only one T-rich sequence was chosen because of T-rich sequences tend to from non-rigid tertiary structures and the chance of being aptamer was thought to be very low. As shown in Figure 2c, C14B can bind AGR2 with the highest affinity (*K_d_* = 13.1±7.2 nM). Titration of C14B to GST-beads revealed no observable binding, establishing that the binding target of C14B was indeed AGR2. Other three sequences have similar binding constants to AGR2 but a little weaker than C14B, in which the *K_d_* of C14A is 20.9±5.2 nM, C14C is 44.6±7.0 nM and C14D is 48.4±15.6 nM. (Figure S2).

### Sequence optimization of aptamer C14B

Since C14B is the best aptamer we obtained from the four sequences tested, it was choses for future optimization and characterization. The length of C14B is 87mer, which is disadvantageous for future applications because it is inconvenient and expensive to synthesize such a long sequence. To identify the binding region of the aptamer, the marginal sequences of C14Bwas gradually truncated. Two truncated sequencesC14B0 and C14B1 from C14B were shown in Table 1. Subsequent binding affinity experiments revealed that both C14B0 (8.5±3.6 nM) and C14B1 (19.1±5.1 nM) have similar *K_d_* to AGR2 as that of original C14B (13.1±7.2 nM). The majority of eliminated sequences were primer sequences, implying that the binding region of the aptamer was the middle of random sequences and the primer sequences do not or contribute little to the binding affinity of aptamer. C14B1 has five portions of poly-G and we designed five truncated sequences by removing one of poly-G portion each (Table S2). Removal of any poly-G portion would destroy its binding affinity to certain extend (Figure S3), suggesting the whole G-motif is required for aptamer binding. Taking together, these results indicate that C14B1, which was only 33 mer, was the essential binding region to AGR2. Thus, C14B1 was applied for further characterization and probe design.

**Table 1:** The sequences of C14B, C14B0 andC14B1, and their Kd to AGR2.

Aptamer	Sequences	Kds
**C14B**	TCTCGGACGCGTGTGGT**CGGGTGGGAGTTGTGGGGGGGGGTGGGAGGGTT**CTTTGTTTGATCTTTCTCGCTGCCTGGCCCTAGAGTG	K_d_ = 13.1±7.2 nM
**C14B0**	**CGGGTGGGAGTTGTGGGGGGGGGTGGGAGGGTT**CTTTGTTTGATCTTTCTCGCTGCCTGGCCCTAGAGTG	K_d_ = 8.5±3.6 nM
**C14B1**	**CGGGTGGGAGTTGTGGGGGGGGGTGGGAGGGTT**	K_d_ = 19.1±5.1 nM

### Selectivity of Aptamer C14B1

**Figure 3:**
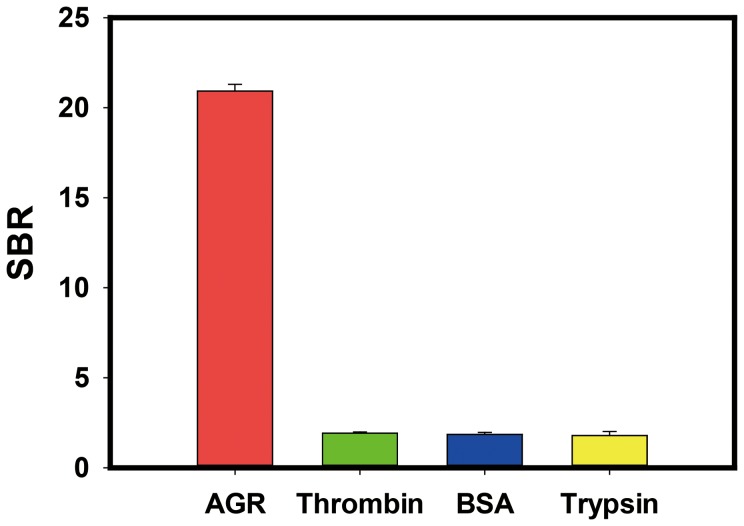
Specificity of aptamer C14B1 against AGR2 and three control proteins, BSA, Trypsin and Thrombin. The signal-to-background ratio (SBR) is defined as the fluorescence signal ratio of FAM-labeled aptamer to FAM-labeled random sequence when treating with protein modified beads. The data were the average of triplicate experiment results.

In order to demonstrate the specific interaction between AGR2 and C14B1, three control proteins BSA, thrombin and trypsin were coupled with the NHS-sepharose-beads and then were incubated with C14B1. As shown in Figure 3, C14B1 could bind to target AGR2 strongly, while there were no or little binding affinity towards thrombin, BSA and trypsin, demonstrating the high selectivity of the aptamer.

### Aptamer C14B1 is an Intramolecular Parallel G-quadruplex

Further examination revealed that C14B1 has multiple stretches of guanines (C**GGG**T**GGG**AGTTGT**GGGGGGGGG**T**GGG**A**GGG**T). It is well-known that guanine-rich sequences can fold into four-stranded secondary structures called quadruplexes. According to G-Quadruplex prediction formula d(G_3+_N_1-7_G_3+_N_1-7_G_3+_N_1-7_G_3+_) [45], C14B1 had great probability to form a G-Quadruplex structure. In the G-quadruplex, the planar square arrangement of four guanines (tetrad) is stabilized by Hoogsteen hydrogen bonding. Depending on the direction of the strands or parts of a strand that form the tetrads, structures may be described as parallel or antiparallel. Moreover, these quadruplexes can form through the intermolecular association of four or two DNA molecules, or by the intramolecular folding of a single strand containing four blocks of guanines [46] CD experiments were performed to investigate the secondary structure of aptamerC14B1. The CD spectrum of C14B1 showed a negative peak near 240 nm and a positive peak near 260 nm, a typical spectrum of a parallel G-quadruplex [47]. In order to investigate whether C14B1 forms an intermolecular 4 stranded or intramolecular single stranded G-quadruplex structure, the dependence of melting temperature on the concentration of C14B1 was studied [48]. The melting temperature of C14B1 was found to be 59°C, which was independent of oligonucleotide concentration (Figure S4), indicating that the aptamer forms an intramolecular G-quadruplex.

### Binding Affinity and Structure Stability of C14B1 is K^+^ Dependent

**Figure 4:**
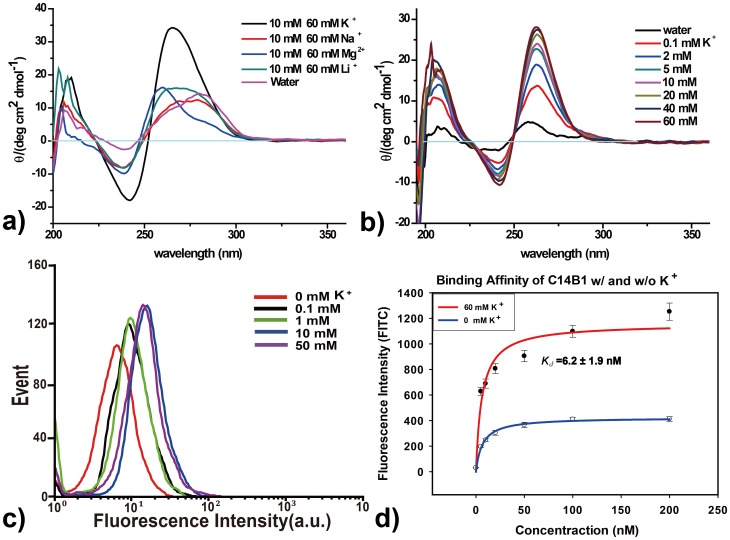
Characterization of the aptamer C14B1. a) CD spectra of the aptamer C14B1 in the presence of different cations. This aptamer has a positive band near 260 nm that can be assigned to a parallel quadruplex structures. b)The structural stability of the aptamer relies heavily on [K^+^]. c) Fluorescent signal migration of C14B1 binding to AGR2-beads at different concentration of K^+^ was investigated. d) The binding constants of C14B1 in the buffer w/ and w/o K^+^. The data were the average of triplicate experiment results.

Many DNA aptamers have been found to form G-quadruplex structures [49]–[51]. It is well established that the existence of a monovalent cation (especially potassium) in the center of these tetrads can significantly stabilize G-quadruplexes [52]–[54]. Thus, we investigated how a cation would affect the structural stability of C14B1 and its binding activity towards to AGR2. As shown in Figure 4a, the stability of the C14B1 G-quadruplex structure is strongly dependent on the presence of the monovalent ion. With 60 mM of KCl, strong CD peaks were observed, suggesting the formation of stable G-quadruplex structure. Replacing KCl with the same concentration of LiCl, NaCl, and MgCl_2_ led to a dramatic intensity decrease in CD.

We further studied the effect of K^+^ concentration on the stability of G-quadruplex. In Figure 4b, addition of 0.1 mM K^+^ in phosphate buffer dramatically increased the CD intensity at 240 and 260 nm. CD absorption intensity enhanced with the increase of K^+^ concentration and reached a plateau when K^+^ concentration was higher than 20 mM. The binding affinity of C14B1 at different concentrations of K^+^ was also investigated. As the flow cytometry results shown in Figure 4c, very weak binding of C14B1 towards AGR2-beads was observed when there was no K^+^ in the buffer, and the binding affinity kept increasing with the addition of K^+^. The binding constants of C14B1 were measured and compared in the presence or absence of K^+^ (Figure 4d). The *K_d_* value in the presence of K^+^ was determined to be 6.2±1.9 nM. The results demonstrated that formation of G-quadruplex is important for the binding capability of aptamer C14B1 to its target, which is highly K^+^ dependent.

### Allosteric molecular beacons for detection AGR2

**Figure 5:**
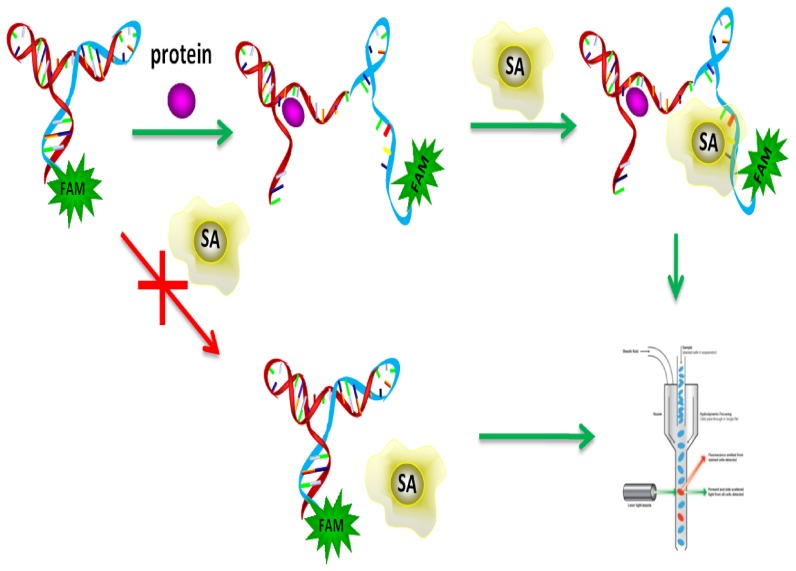
Working principle of allosteric molecular beacon probe for sensitive and selective detection of AGR2 based on fluorescent flow cytometry analysis.

Clinical studies have already shown that the AGR2 protein is overexpressed in a wide range of human cancers. The sensitive and selective detection of AGR2 is thus of great importance to early cancer diagnostics. Herein, by applying the selected and optimized aptamer C14B1, we designed and developed an allosteric molecular beacon against AGR2, named AGR2-aMB, which converts the molecule recognition property of aptamer to fluorescence flow cytometry signal for AGR2 sensing [55]. Figure 5 illustrates the working principle of AGR2-aMB. An AGR2-aMB is a ssDNA consisting of an streptavidin (SA) aptamer sequence [56], a C14B1 sequence, a short sequence complimentary to a small part of the SA aptamer sequence, and a fluorophore. A stable hairpin structure is formed by intramolecular hybridization between the SA aptamer sequence and the complementary sequence, temporarily disabling the probe's ability to bind with SA beads. Consequently, when incubated with SA beads, no probe can bind to SA beads, and the beads display very low fluorescence. In the presence of AGR2, however, the C14B1 sequence in the loop of aMB binds to the target sequence, which in turn disrupts the hairpin structure to free the SA binding sequence, thereby activating the probe's binding affinity to SA beads. Thus, the AGR2-bound probe will bind to SA beads, which will fluoresce strongly because the probe is FAM labeled. Target molecules can be detected and quantified by reading the fluorescence intensity of SA beads through flow cytometer.

**Figure 6:**
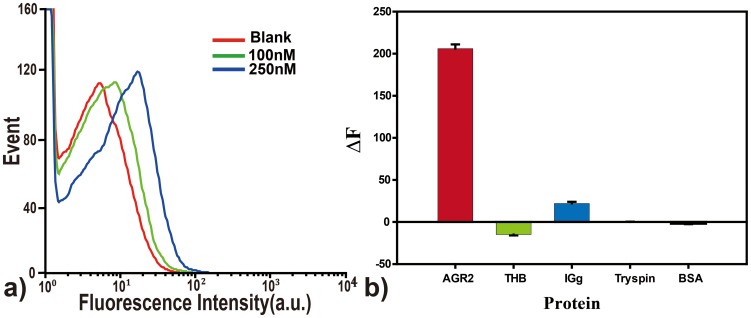
Evaluation of the performance of AGR2-aMB. a) Response of AGR2-aMB to different concentrations of AGR2 in Tris-HCl buffer. b) Response of AGR2-aMB to different targets including AGR2 (100 nM), BSA, IgG, Trypsin and Thrombin (500 nM each for control proteins). The data were the average of triplicate experiment results.

We designed AGR2-aMB, with the following sequence: **CGACGCACCGATCGCAGGTTCGGGA**TTTTCGGGTGGGAGTTGTGGGGGGGGGTGGGAGGGTT -FAM, where the AGR2 binding region is underlined and the SA aptamer sequence is in bold. Stable hairpin structure is formed by intramolecular hybridization (Figure S5). In our experiment, when only AGR2-aMB incubated with SA beads, no probe can bind to SA beads, and the beads fluoresced very weakly. In the presence of AGR2, however, the fluorescence intensity of SA beads increase significantly, suggesting the binding of AGR2-bound probe to SA beads. With the increase of AGR2 concentration, steady increases in fluorescence intensity on the target beads were observed. AGR2 at 100 nM concentration can be easily detected (Figure 6a). A series of proteins including BSA, trypsin, thrombin, and IgG were employed as controls (500 nM), and no distinct fluorescent signals were observed (Figure 6b), indicating an excellent specificity of the AGR2-aMB towards target molecule. The results demonstrated the sensitivity and specificity of the allosteric molecular beacon probe, implying its potential for application in real sample analysis, such as protein function study and disease diagnosis.

In conclusion, we have developed new aptamer probes for specific recognition of the AGR2. In the SELEX process, AGR2-GST fused protein was used as the target protein, and linked to sepharose beads through the mild, yet specific, noncovalent GST-Glutathione interaction. Beads-based SELEX allowed the use of simple, yet effective, flow cytometry analysis to monitor the progress of the selection, avoiding the tedious, time consuming and radioactive EMSA process. Through multiple rounds of selection with GST as a control, we have identified aptamers that selectively recognize AGR2 with nanomolar *K_d_* values. CD measurements and melting-temperature assays demonstrated that the optimal aptamer C14B1 forms an intramolecular parallel G-quadruplex structure and its structure and binding affinity are strongly dependent on the nature of the monovalent ion. Furthermore, with our design of AGR2-aMB, AGR2 could be sensitively and selectively detected The aptamer sequences and sensors reported here has great potential to serve as a useful tool for early diagnosis and prognosis of cancer and for fundamental research to elucidate the biochemical functions of AGR2.

## Materials and Methods

### Initial library design

The HPLC-purified library containing a central randomized sequence of 45 nucleotides flanked by two 20-nt and 22-nt primer hybridization sites. Initial library was: 5′-TCT CGG ACG CGT GTG GTC GG-N45-C TCG CTG CCT GGC CCT AGA GTG-3′, forward primer: 5′-FAM-TCT CGG ACG CGT GTG GTC GG-3′, reverse primer: 5′-Biotin-CAC TCT AGG GCC AGG CAG CGA G-3′.

### Preparation of AGR2-GST fused protein

The plasmid pGEX-GST-AGR2 was transformed into the engineering strain BL-21 and the GST-tagged AGR2 protein was expressed. After purification with glutathione sepharose beads (GE healthcare) by affinity chromatography, the GST-AGR2 fused protein was linked to sepharose beads for positive selection. After being washed several times with W1 buffer (25 mM Tris-HCl pH 7.5, 150 mM NaCl, 0.07% β-mercaptoethanol, 1% Triton X-100), the final purified AGR2-GST-beads were stored in sterilized PBS buffer. Flow cytometry analysis with TAMRA-labeled anti-AGR2 monoclonal antibody (Santa Cruz) and SDS-PAGE indicated that the GST-AGR2 fused protein was successfully linked to the sepharose beads.

### Selection of AGR2 binding aptamers

The procedures of selection were as follows. The ssDNA pool (200 pmol) dissolved in binding buffer (200 µL, 1×PBS buffer containing 137 mM NaCl, 2.7 mM KCl, 10 mM Na_2_HPO_4_ and 2.0 mM KH_2_PO_4_, pH 7.4) was denatured by heating at 95°C for 5 min, then quickly cooled on ice for 10 min, and subsequently incubated for another 10 min at room temperature before binding. The ssDNA pool was then incubated with negative GST beads (1.0×10^5^ beads) for counter selection to remove sequences non-specifically binding to GST beads. After filtering with a homemade filter column, the filtrate was incubated with positive AGR2-GST-beads (1.0×10^5^ beads) at 37°C for 45 min. The unbound or nonspecifically bound oligoes were removed by filtration. The sequences bound to the target-coated beads were then amplified by PCR with FAM and biotin-labeled primers (5–15 cycles of 0.5 min at 94°C, 0.5 min at 53°C, and 0.5 min at 72°C, followed by 5 min at 72°C; the Easytaq plus polymerase and dNTPs were obtained from Transgen Beijing). After denaturation in NaOH (0.1 M), the selected sense ssDNA was separated from the biotinylated antisense ssDNA strand on streptavidin-coated sepharose beads (GE healthcare) and used for next round selection. For the first-round selection, the amount of initial ssDNA pool was 5 nmol, dissolved in 500 µL binding buffer, and the counter-selection step was eliminated. To acquire aptamers with high affinity and specificity, the selection strength was enhanced gradually by increasing the number of washes (from three to ten times with 200 µL 1×PBS buffer each) and decreasing the amount of the ssDNA library per round (from 200 to 150 pmol).

### Cloning and DNA sequencing

The resulting pool from the 14^th^ round was PCR amplified, cloned and sequenced (Shanghai Sangon sequencing facility). The resulting 62 sequences were subjected to multiple sequence alignment analysis by using Clustal W 6.0 software to discover highly conserved motifs in groups of selected DNA sequences. The discovered consensus sequences with high repeats among selected pools were then chemically synthesized for further testing.

### Flow cytometric analysis

To monitor the enrichment of aptamers after selection, the FAM-labeled ssDNA pool was incubated with 5×10^4^ AGR2-GST-beads or GST-beads in binding buffer (200 µL) at 37°C for 45 min. Beads were washed three times with 200 µL binding buffer by means of filtration, and suspended in binding buffer (250 µL). The fluorescence intensity of the resulting beads was monitored with a FACSAria cytometer (Becton Dickinson Immuno cytometry systems) by counting 10000 events. The binding affinities of aptamers were determined by incubating AGR2-GST-beads (5×10^4^) with various concentrations of FAM-labeled aptamers (pre-heat-treated) in binding buffer (200 µL) at 37°C for 45 min in the dark. Beads were then washed three times with the binding buffer, then resuspended in binding buffer (250 µL) and subjected to flow cytometry analysis. The FAM-labeled unselected ssDNA library was used as negative control for the nonspecific binding evaluation. All binding experiments were repeated two to four times. The mean fluorescence intensity of target protein labeled by aptamers was used to evaluate binding affinity by subtracting the mean fluorescence intensity of nonspecific binding produced by unselected ssDNA library. The dissociation constants (*K_d_*) of the fluorescent ligands were obtained by fitting the dependence of fluorescence intensity of specific binding on the concentration of the ligands to the equation (1): Y = B_max_X/(*K_d_*+X) using SigmaPlot software.

### Circular dichroism spectroscopy

CD measurements were carried out on a Jasco J-810 spectropolarimeter equipped with a programmable temperature-control unit (Julabo HP-4). The concentration of DNA samples were 2 mM. Before the CD spectrum measurement, the DNA samples were annealed by heating to 95°C for 5 min, then rapidly cooled on ice for 10 min, and incubated for another 10 min at room temperature. The spectra from 400 to 200 nm were obtained by using 1 nm slit width and 0.1 nm scanning resolution. Each CD spectrum was an average of 8 scans with the buffer background subtracted.

### UV thermal-denaturation experiment

UV absorbance and melting studies were carried out on an Agilent 8453 spectrophotometer equipped with a programmable temperature-control unit (Agilent 89090A). Melting temperatures (T_m_) were taken as the temperature of half-dissociation of the quadruplex and were obtained from the maximum of the first derivative dA/dT plots at 295 nm. The heat-treated DNA solutions at several concentrations were introduced into a quartz cuvette and overlaid with a thin layer of silicone oil to prevent evaporation. The optical path length was 1 cm. Absorbance and temperature were recorded every 2°C.

### Evaluation of AGR2-aMB

AGR2-aMB (40 nM) was annealed by heating to 95°C for 5 minutes in 200 µL Tris-HCl buffer (25 mM Tris-HCl, 120 mM NaCl, 0.5 mM KCl, 2 mM MgCl_2_ at pH 7.4), and then rapidly cooled on ice for 10 min, and subsequently waiting for another 10 min at room temperature before use. To the solution of AGR2-aMB, various concentrations of AGR2 were added and the resulting solutions were allowed to incubate at 37°C for 30 min. To the mixture, 1 µL of streptavidin beads (about 5×10^4^ beads) were added and the mixture was allowed to set for 45 min in dark. After washing twice with Tris-HCl buffer, SA beads were filtered to eliminate unbound AGR2-aMB and resuspended in Tris-HCl buffer solution 250 µL before flow cytometry analysis. In selectivity tests, BSA (Tagene Biotechnology Xiamen), trypsin (Dingguo Beijing), thrombin (alfa) and IgG (Dingguo Beijing) were used.

## Supporting Information

Figure S1 **The sequences of 62 clones.** One subfamily is guanosine-rich sequences (22 clones), and the other is thymine-rich sequences (40 clones).
(TIF)

Figure S2 **The dissociation constant measurement of C14A, C14B, C14C and C14D against AGR2 GST and GSH.**
(TIF)

Figure S3 **Flow cytometry assay to monitor the binding of C14B1 and its five truncated sequences with a) AGR2 (target protein) and b) GST (control protein).**
(TIF)

Figure S4 **UV thermal-denaturation experiment of C14B1.** Denaturation profiles obtained at 295 nm for the aptamer at three different concentrations (2 µM, 4 µM, 8 µM). The T_m_ (59°C) at 295 nm is independent of oligonucleotide concentration, indicating that the aptamer forms an intramolecular G-quartet.
(TIF)

Figure S5 **The secondary structure of AGR-aMB.** Stable hairpin structure is formed by intramolecular hybridization between the SA aptamer sequence and the complementary sequence of C14B_1_.
(TIF)

Table S1 **The sequences of C14A, C14B, C14C and C14D, and their K_d_ to AGR2.**
(DOC)

Table S2 **The truncated sequences by removing one of poly-G portion each from C14B1.**
(DOCX)
