# Supplementary figures and images for: Identification, Characterization and Application of a G-Quadruplex Structured DNA Aptamer against Cancer Biomarker Protein Anterior Gradient Homolog 2
Source: PLoS One. 2012 Sep 28;7(9):e46393. doi: 10.1371/journal.pone.0046393 (PMC3460915; doi:10.1371/journal.pone.0046393)

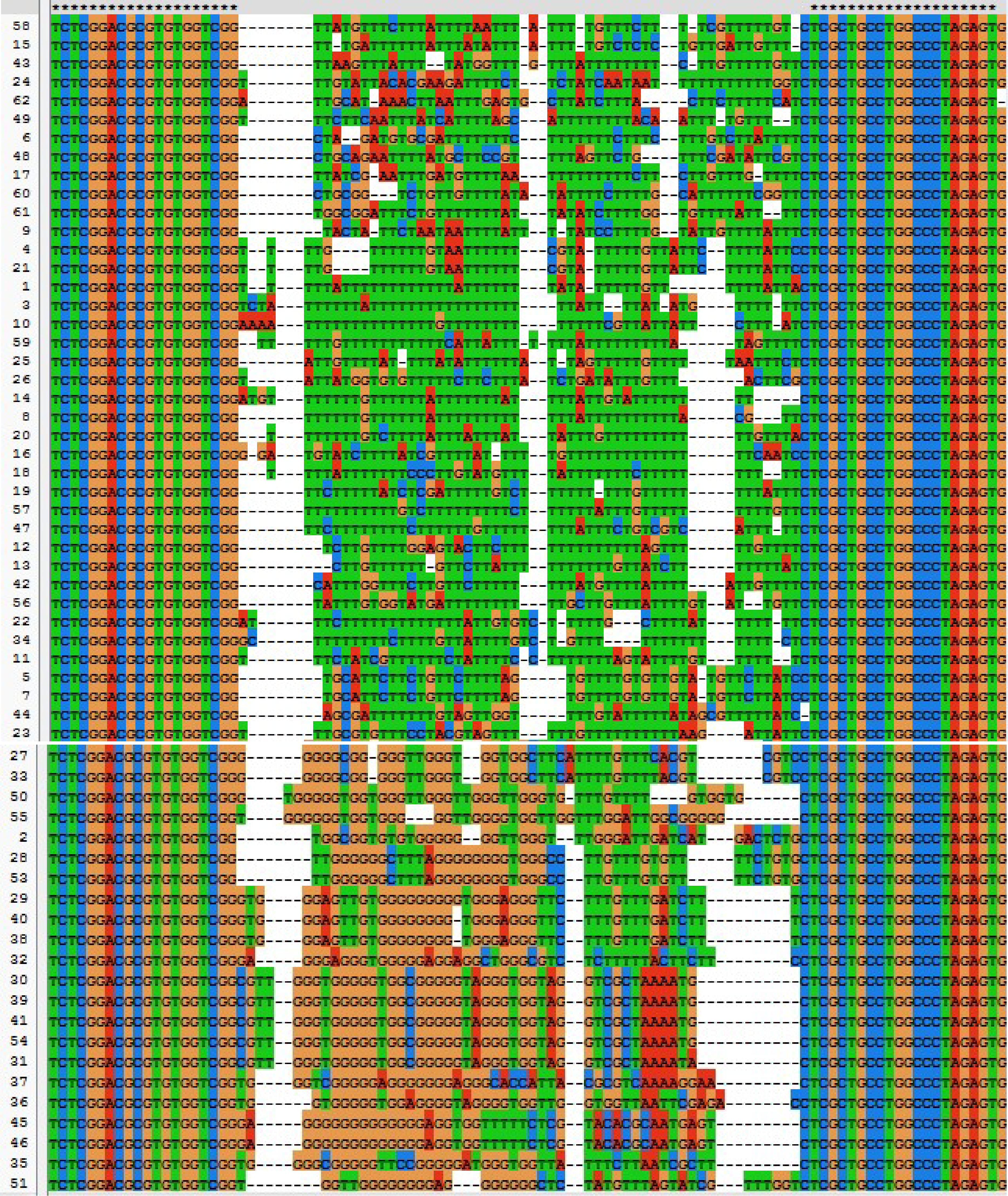

Supplement: Figure S1 — The sequences of 62 clones. One subfamily is guanosine-rich sequences (22 clones), and the other is thymine-rich sequences (40 clones). (TIF) [file pone.0046393.s001.tif]

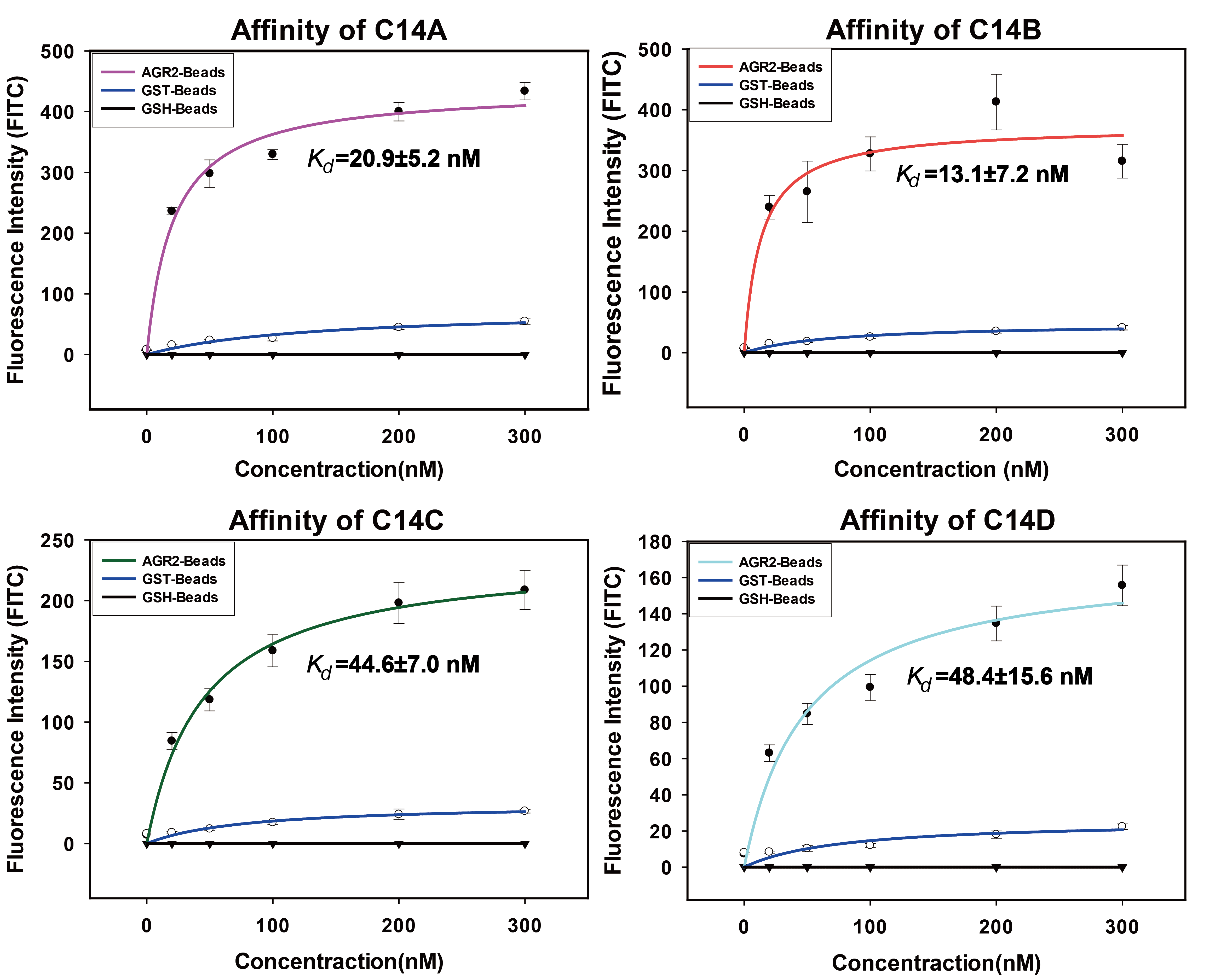

Supplement: Figure S2 — The dissociation constant measurement of C14A, C14B, C14C and C14D against AGR2 GST and GSH. (TIF) [file pone.0046393.s002.tif]

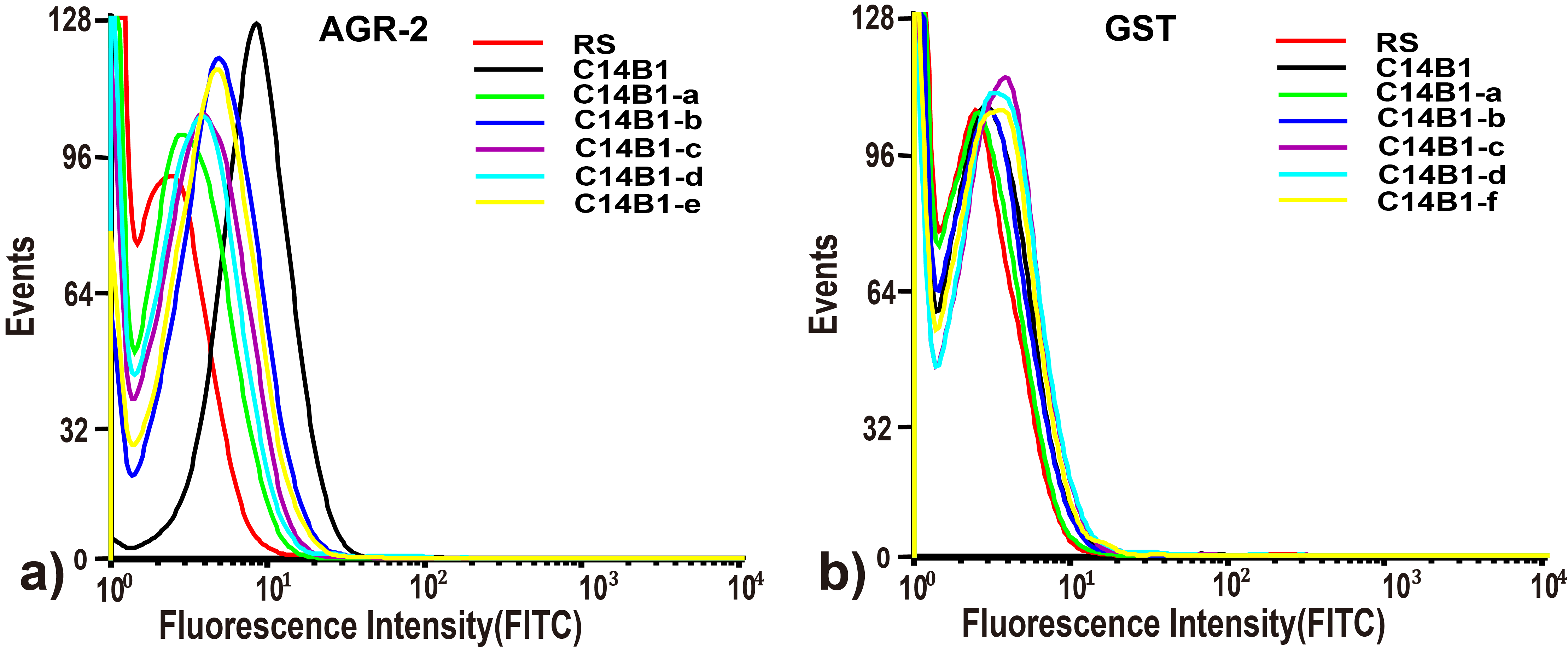

Supplement: Figure S3 — Flow cytometry assay to monitor the binding of C14B1 and its five truncated sequences with a) AGR2 (target protein) and b) GST (control protein). (TIF) [file pone.0046393.s003.tif]

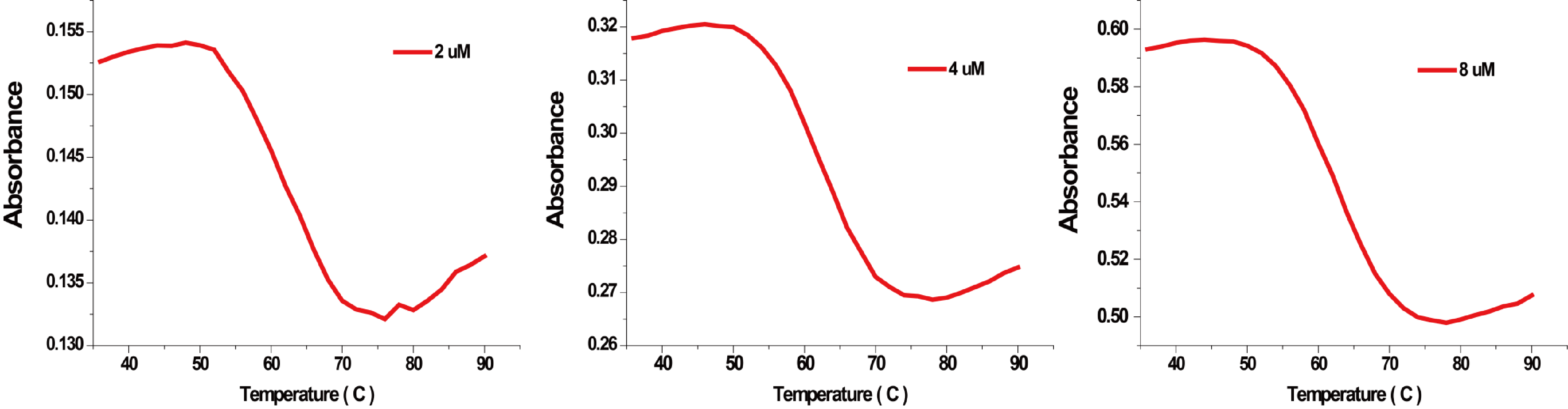

Supplement: Figure S4 — UV thermal-denaturation experiment of C14B1. Denaturation profiles obtained at 295 nm for the aptamer at three different concentrations (2 µM, 4 µM, 8 µM). The Tm (59°C) at 295 nm is independent of oligonucleotide concentration, indicating that the aptamer forms an intramolecular G-quartet. (TIF) [file pone.0046393.s004.tif]

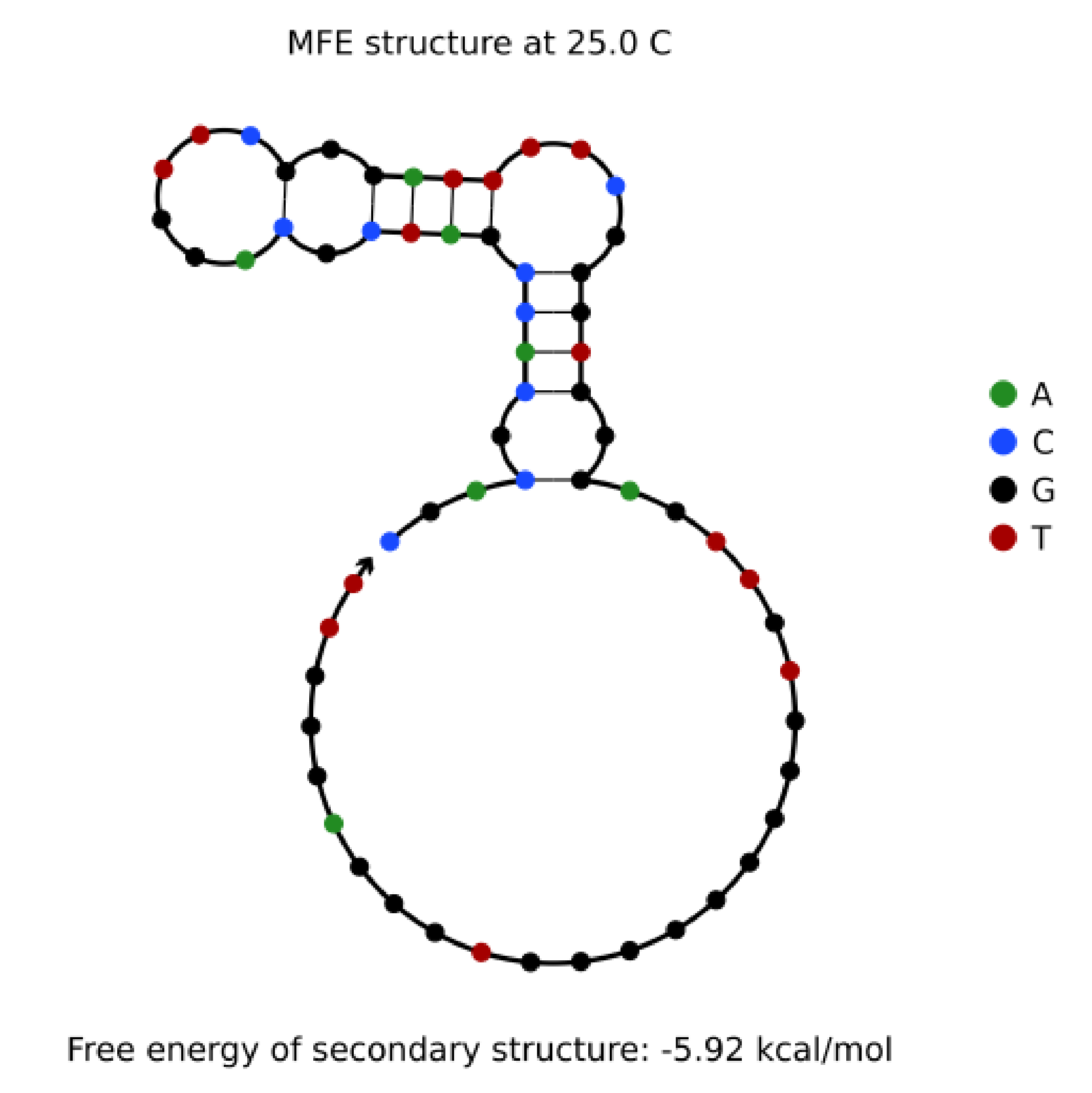

Supplement: Figure S5 — The secondary structure of AGR-aMB. Stable hairpin structure is formed by intramolecular hybridization between the SA aptamer sequence and the complementary sequence of C14B1. (TIF) [file pone.0046393.s005.tif]
