# Supplementary material for: Identification, Characterization and Application of a G-Quadruplex Structured DNA Aptamer against Cancer Biomarker Protein Anterior Gradient Homolog 2
Source: PLoS One. 2012 Sep 28;7(9):e46393. doi: 10.1371/journal.pone.0046393 (PMC3460915; doi:10.1371/journal.pone.0046393)
Supplement: Table S1 — The sequences of C14A, C14B, C14C and C14D, and their Kd to AGR2. (DOC) [file pone.0046393.s006.doc]

**Table S1.The sequences of C14A, C14B, C14C and C14D, and their K_d_ to AGR2.**

| **Aptamer** | **Sequences** | **K_d_** |
| --- | --- | --- |
| **C14A** | TCTCGGACGCGTGTGGTCGGGGGGGCGGGGGTTGGGTGGTGGCTTCATTTTGTTTCACGTCGTCCTCGCTGCCTGGCCCTAGAGTG | K_d_=20.9±5.2 nM |
| **C14B** | TCTCGGACGCGTGTGGTCGGGTGGGAGTTGTGGGGGGGGGTGGGAGGGTTCTTTGTTTGATCTTTCTCGCTGCCTGGCCCTAGAGT | K_d_=13.1±7.2 nM |
| **C14C** | TCTCGGACGCGTGTGGTCGGCGTTGGGTGGGGGTGGCGGGGGTAGGGTGGTAGGTCGCTAAAATGCTCGCTGCCTGGCCCTAGAGTG | K_d_=44.6±7.0 nM |
| **C14D** | TCTCGGACGCGTGTGGTCGGTGCATTCTTCTGTTCTTTTAGTGTTTTGTGTTGTATGTTCTTATCCTCGCTGCCTGGCCCTAGAGTG | K_d_=48.4±15.6 nM |
