# Supplementary material for: Identification, Characterization and Application of a G-Quadruplex Structured DNA Aptamer against Cancer Biomarker Protein Anterior Gradient Homolog 2
Source: PLoS One. 2012 Sep 28;7(9):e46393. doi: 10.1371/journal.pone.0046393 (PMC3460915; doi:10.1371/journal.pone.0046393)
Supplement: Table S2 — The truncated sequences by removing one of poly-G portion each from C14B1. (DOCX) [file pone.0046393.s007.docx]

**Table S2.The truncated sequences by removing one of poly-G portion each from C14B1.**

| **Name** | **Sequence** |
| --- | --- |
| **C14B1** | 5'-CGGGTGGGAGTTGTGGGGGGGGGTGGGAGGGTT-FAM-3' |
| **C14B1-a** | 5'-CGGGTGGGAGTTGT~~GGGGGGGGG~~TGGGAGGGTT-FAM-3' |
| **C14B1-b** | 5'-C~~GGG~~TGGGAGTTGTGGGGGGGGGTGGGAGGGTT-FAM-3' |
| **C14B1-c** | 5'-CGGGT~~GGG~~AGTTGTGGGGGGGGGTGGGAGGGTT-FAM-3' |
| **C14B1-d** | 5'-CGGGTGGGAGTTGTGGGGGGGGGT~~GGG~~AGGGTT-FAM-3' |
| **C14B1-e** | 5'-CGGGTGGGAGTTGTGGGGGGGGGTGGGA~~GGG~~TT-FAM-3' |
